# Eosinophilic Ureteritis in a Child With Ureteropelvic Junction Obstruction and Vesicoureteral Reflux

**DOI:** 10.7759/cureus.9214

**Published:** 2020-07-15

**Authors:** Gavin Stormont, John Makari, Jiri B Bedrnicek, Claudia Berrondo

**Affiliations:** 1 Urology, University of Nebraska Medical Center, Omaha, USA; 2 Urology/Pediatric Urology, Children's Hospital and Medical Center, Omaha, USA; 3 Pathology, Children's Hospital and Medical Center, Omaha, USA; 4 Surgery/Pediatric Urology, University of Nebraska Medical Center, Omaha, USA; 5 Pediatric Urology, Children’s Hospital and Medical Center, Omaha, USA

**Keywords:** ureteropelvic junction obstruction, eosinophilic ureteritis, vesicoureteral reflux

## Abstract

Eosinophilic ureteritis is a rare cause of ureteral obstruction, and to date the diagnosis can only be made on pathologic examination. The true underlying cause is not well understood, but there may be some association with eosinophilia, atopy and/or trauma. We present a case of a two-year-old boy with ureteropelvic junction obstruction (UPJO) and ipsilateral vesicoureteral reflux (VUR) found to have eosinophilic ureteritis. To our knowledge, this is the youngest reported patient with this finding, and the only patient with eosinophilic ureteritis causing UPJO with concomitant VUR.

## Introduction

Ureteropelvic junction obstruction (UPJO) is the most common cause of antenatally diagnosed pathologic hydronephrosis accounting for 11% of cases. UPJO is more common in boys than girls with a ratio of 2:1 and occurs on the left side in two-thirds of cases [1]. The pathophysiology of UPJO can be divided into intrinsic and extrinsic causes. The most common intrinsic causes are scarring of ureteric valves and ureteral hypoplasia [2,3]. Another well reported but rare cause of UPJO is fibroepithelial polyps [4]. The most common causes of extrinsic UPJO are lower pole crossing renal vessel(s), congenital abnormalities such as horseshoe kidney and duplication abnormalities, and scar from previous surgery [5,6]. We report a case of eosinophilic pyeloureteritis in a child with vesicoureteral reflux (VUR) and UPJO. To our knowledge, this is the only reported case of eosinophilic ureteritis causing UPJO with associated VUR, and our patient is the youngest to be reported in the literature with this pathologic finding.

## Case presentation

**Figure 1:**
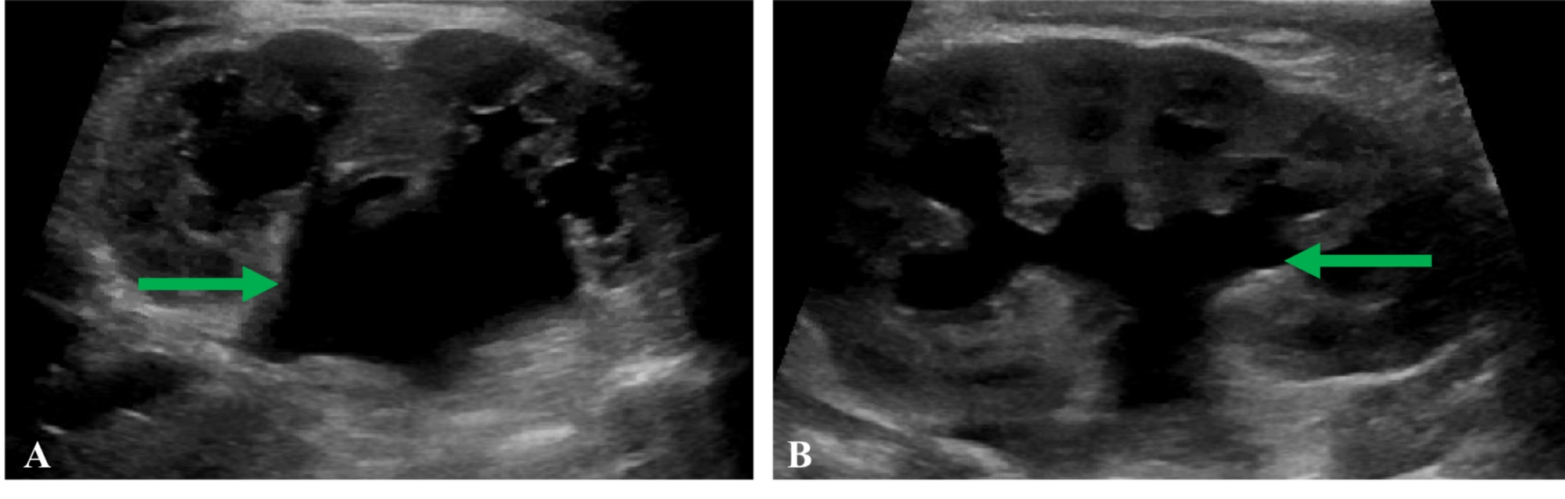
Initial renal ultrasound demonstrating bilateral moderate hydronephrosis (A) Right kidney with moderate hydronephrosis (green arrow). (B) Left kidney with moderate hydronephrosis (green arrow).

An 11-day-old term male presented to pediatric urology with antenatally diagnosed bilateral hydronephrosis. Renal ultrasound after birth at 11 days of age revealed moderate left hydronephrosis and moderate right hydronephrosis with ureteral dilation (Figure 1). 

**Figure 2:**
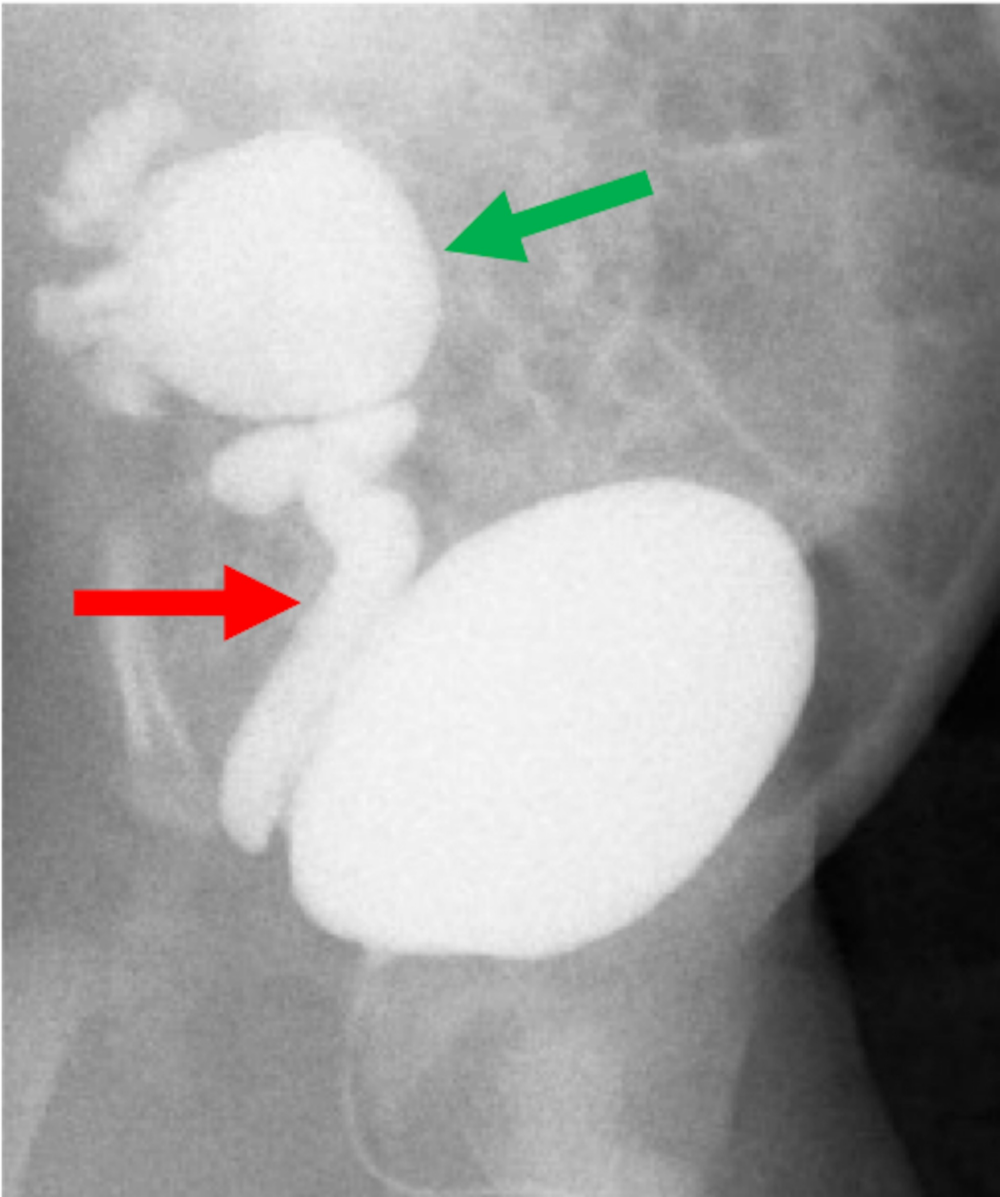
Initial voiding cystourethrogram Voiding cystourethrogram demonstrating right grade 5 vesicoureteral reflux. The green arrow points to the dilated right renal pelvis, and the red arrow points to the dilated right ureter.

Further evaluation with voiding cystourethrogram (VCUG) demonstrated right grade 5 VUR with a concern for possible secondary UPJO (Figure 2). 

**Figure 3:**
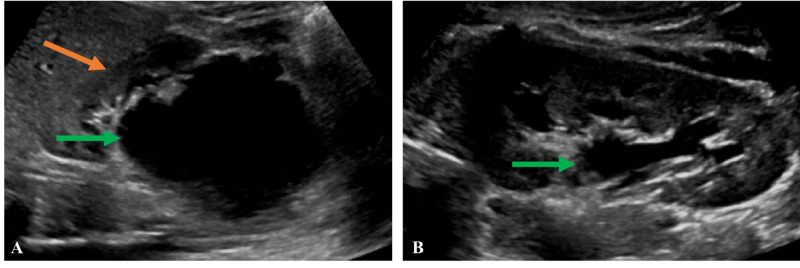
Follow-up renal ultrasound (A) Right kidney with severe hydronephrosis (green arrow) with associated parenchymal thinning and echogenic renal parenchyma (orange arrow). (B) Left kidney with stable moderate hydronephrosis (green arrow).

He was initially managed with prophylactic antibiotics and serial renal ultrasounds. Ultrasound at 8 and 14 months of age demonstrated stable bilateral hydronephrosis. At 27 months of age, follow-up renal ultrasound demonstrated worsening right hydronephrosis with parenchymal thinning and abnormal echogenic renal parenchyma (Figure 3). 

**Figure 4:**
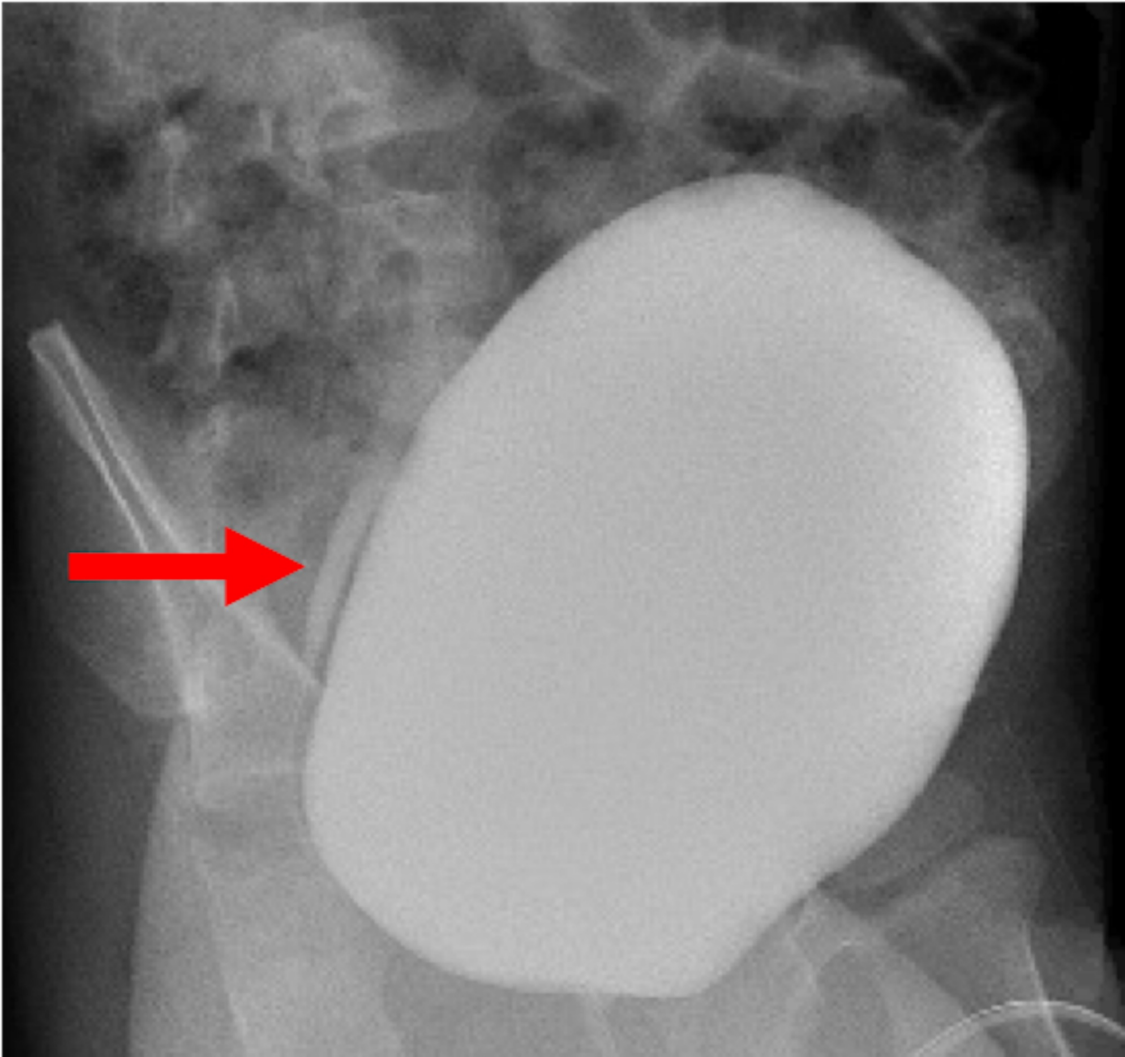
Follow-up voiding cystourethrogram Voiding cystourethrogram demonstrating right grade 1 vesicoureteral reflux. The red arrow points to the right ureter.

At 31 months of age, he underwent follow-up VCUG, which demonstrated residual right grade 1 VUR, and a renogram, which demonstrated delayed drainage of the right kidney with 26% differential function, consistent with UPJO (Figure 4). No blood studies were obtained. Based on these findings, the decision was made to proceed with surgical intervention. 

**Figure 5:**
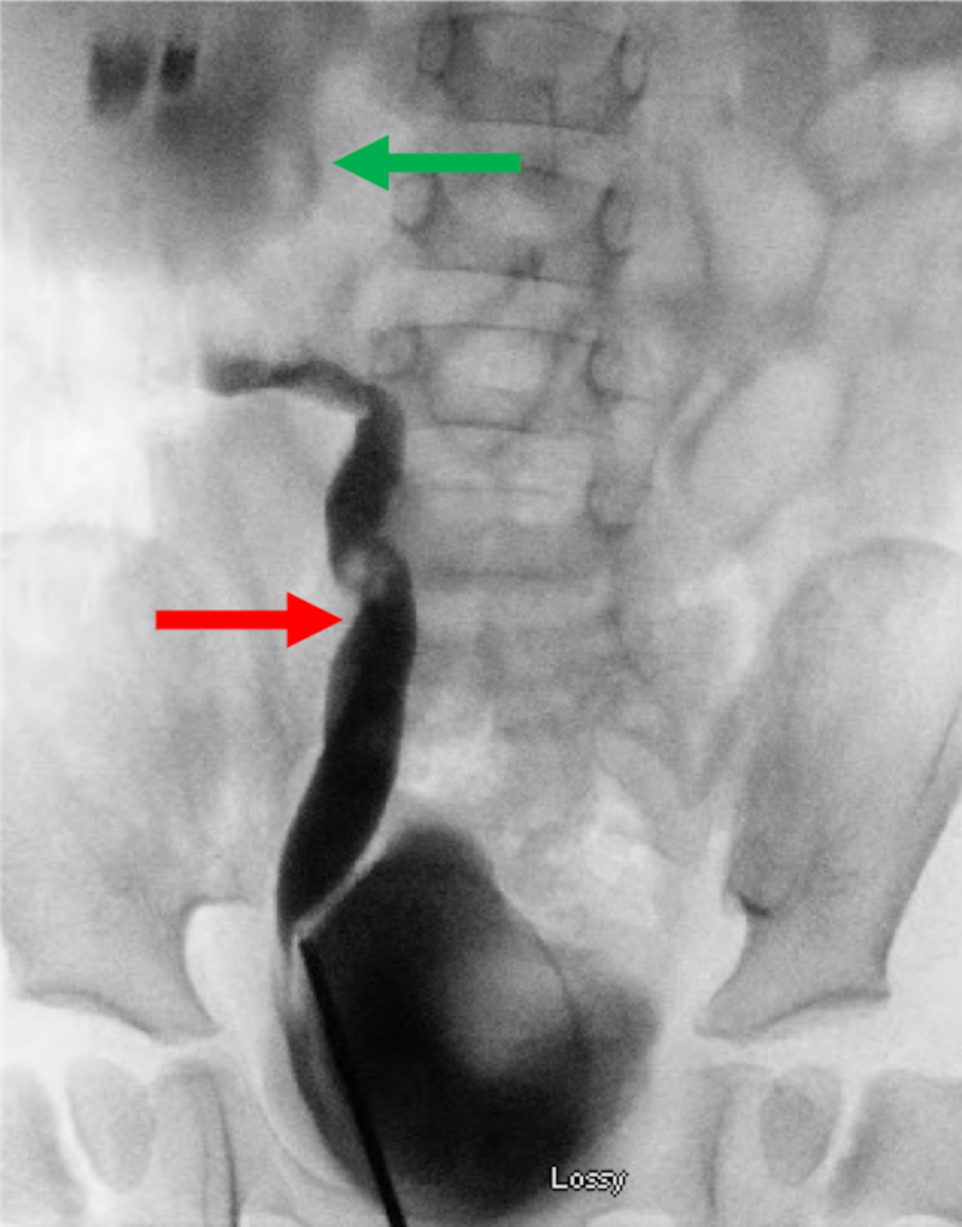
Intraoperative right retrograde pyelogram Intraoperative right retrograde pyelogram demonstrating a dilated right ureter (red arrow) and obstruction at the ureteropelvic junction and a severely dilated right renal pelvis (green arrow).

He underwent surgical repair with an open right dismembered pyeloplasty at 33 months of age. Initial cystoscopy revealed a normal bladder with normal bilateral orthotopic ureteral orifices. Right retrograde pyeloureterogram revealed a dilated ureter with stenosis at the ureteropelvic junction (UPJ) with severe hydronephrosis (Figure 5). Intraoperatively, there was no extrinsic compression of the UPJ. The stenotic UPJ was identified and excised, and the ureter was anastomosed to the renal pelvis. There was no visible lesion noted grossly on the excised urothelium.

**Figure 6:**
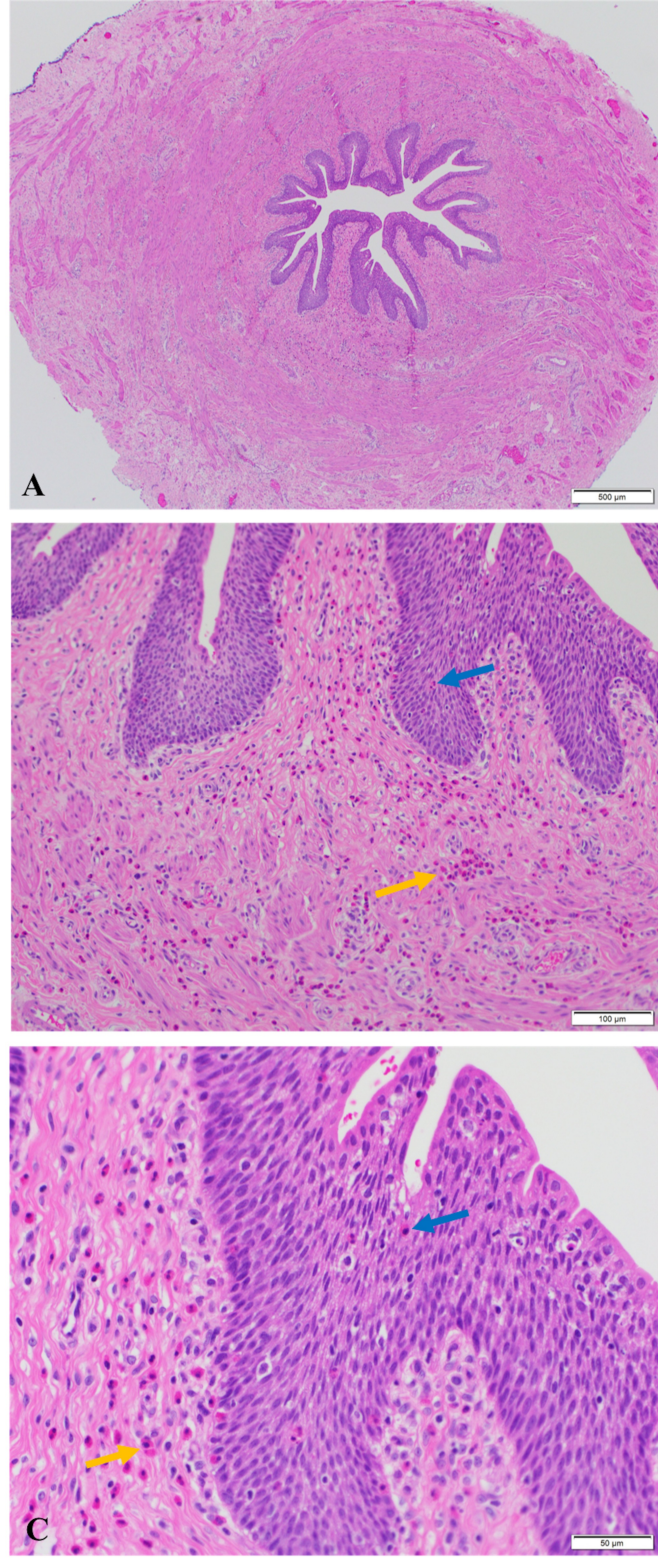
Pathology of the excised ureteropelvic junction (A) Cross section of the ureteropelvic junction. (B) Surgical pathology specimen demonstrates intact ureteral mucosa with eosinophilic infiltrates (blue arrow), and submucosal eosinophilic infiltrates (yellow arrow). (C) Eosinophils infiltrating the ureteral wall (blue arrow) and submucosal ureteral wall (yellow arrow) on high magnification.

His postoperative course was uneventful, and he was discharged home on postoperative day 1. Pathology of the excised segment demonstrated ureteral tissue with subepithelial and epithelial eosinophils (Figure 6).

## Discussion

UPJO is a common cause of antenatally diagnosed hydronephrosis that can be either intrinsic or extrinsic to the ureter [1]. Eosinophilic ureteritis has been reported as a rare cause of ureteral obstruction in the literature with the first reported case in 1979 [7]. Although there are several other reports of eosinophilic ureteritis causing obstruction, to our knowledge this youngest reported patient with this pathology and the only patient with eosinophilic ureteritis causing UPJO with associated VUR.

Several cases of eosinophilic ureteritis have been reported in the literature affecting different portions of the ureter, but little is known about the underlying pathophysiology. Hellstrom et al. first reported the finding of eosinophilic ureteritis in a 21-year-old male with UPJO who developed symptoms about one month after an injury. The authors hypothesized that injury may have been the underlying cause for the eosinophilic ureteritis [7]. Yang and Wu reported a case of a 30-year-old woman with distal ureteral obstruction found to have eosinophilic ureteritis. This patient had no other medical problems or associated injury, but was found to have eosinophilia, decreased complement and IgG, and elevated IgE. The authors hypothesized that the eosinophilic ureteritis may be related to allergy and autoimmune disease [8]. Uyama et al. reported a similar case of a 37-year-old male with distal ureteral obstruction caused by eosinophilic ureteritis with elevated IgE. The pathologic finding was consistent with eosinophilic granuloma [9]. Lumbreras et al. presented a case of a 65-year-old female with arthritis found to have a right proximal ureteral mass thought to be malignant. She did not have eosinophilia. She underwent nephroureterectomy, and the final pathology revealed eosinophilic ureteritis [10]. Spark et al. reported a case of a 44-year-old female with a history of cervical cancer treated with radiation with ureteral obstruction and ulceration found to have eosinophilic ureteritis. She had normal serum studies. They also reported a case of a three-year-old boy with bilateral VUR without obstruction found to have eosinophilic ureteritis of the distal ureter with associated punctate ulcer. He was found to have eosinophilia as well [11].

Eosinophilic ureteritis is a rare and poorly understood entity. The most common presentation is ureteral obstruction, but eosinophilic ureteritis has also been found in the setting of VUR. Because of the variable presentation, the diagnosis can only be made by pathologic exam after surgical excision. There may be some relationship with atopy and/or previous trauma; however not all patients with the finding have a history of allergy/atopy, trauma or associated eosinophilia. There have been no reported cases of recurrence after surgical treatment. In our case, chronic inflammation from VUR may have been the underlying cause of the eosinophilic ureteritis.

## Conclusions

Eosinophilic ureteritis is a rare cause of ureteral obstruction and is currently diagnosed on pathology following surgical intervention. The etiology of the disease remains unknown, but may be associated with allergy/atopy, previous trauma or chronic inflammation. Further investigation is needed to aid in developing diagnostic strategies and potential treatment options.
